# The Motor Neuromuscular Axis: The Overlooked Element of Developmental Programming in Diabetes and Metabolic Syndrome

**DOI:** 10.3390/ijms27136049

**Published:** 2026-07-06

**Authors:** Matheus Felipe Zazula, Stephanie Rubianne Silva Carvalhal, Djennifer T. Maciel, Douglas Moritz, Hellen Yukari Ito Beirauti, Luiza Amorim, Mateus Teixeira da Rocha, Mônica Maciel, Otávio Sales, Paulo Dobgenski, Pedro Braga, Tayná Nery Banckes, Thomas Horlem, Heloísa Deola Confortim, Paulo Ivo Homem de Bittencourt Júnior, Luiz Claudio Fernandes, Katya Naliwaiko

**Affiliations:** 1 Laboratório de Plasticidade Morfofuncional, Departamento de Biologia Celular, Setor de Ciências Biológicas, Centro Politécnico, Universidade Federal do Paraná, Curitiba 81531-908, Paraná, Brazil; djennifermaciel40@gmail.com (D.T.M.); douglasmoritzmoreira@gmail.com (D.M.); yukari.beirauti@gmail.com (H.Y.I.B.); luizaalvesamorim@gmail.com (L.A.); mtr.mateusrocha@gmail.com (M.T.d.R.); monica.mm.maciel@gmail.com (M.M.); otaviosales2005@gmail.com (O.S.); paulinhodobg@gmail.com (P.D.); pedroshbraga@gmail.com (P.B.); taynabanckes@ufpr.br (T.N.B.); heloisa.confortim@ufpr.br (H.D.C.); katya@ufpr.br (K.N.); 2 Laboratório de Fisiologia Celular, Departamento de Fisiologia, Instituto de Ciências Básicas da Saúde, Universidade Federal do Rio Grande do Sul, Porto Alegre 90035-003, Rio Grande do Sul, Brazil; pauloivo@ufrgs.br; 3 Laboratório de Metabolismo Celular, Departamento de Fisiologia, Setor de Ciências Biológicas, Centro Politécnico, Universidade Federal do Paraná, Curitiba 81531-908, Paraná, Brazil; carvalhal.stephanie@gmail.com (S.R.S.C.); tom.horlem@gmail.com (T.H.)

**Keywords:** developmental origins of health and disease, metabolic programming, skeletal muscle metabolism, neuromuscular junction, metabolic flexibility, type 2 diabetes

## Abstract

The Developmental Origins of Health and Disease framework proposes that environmental exposures during critical periods of development can shape physiological systems and influence the risk of chronic diseases later in life, including diabetes and metabolic syndrome. Most research on metabolic programming has focused on classical metabolic organs such as the liver, pancreas, and adipose tissue. However, skeletal muscle plays a central role in systemic glucose homeostasis and metabolic flexibility, accounting for the majority of insulin-stimulated glucose uptake in the body. Because muscle metabolism is closely regulated by neural activity through the organisation of motor units, the development of the motor neuromuscular axis may represent an underexplored dimension of metabolic programming. This review examines evidence linking early-life metabolic environments to neuromuscular development and discusses how alterations in the maturation of motor neurons, neuromuscular junctions, and muscle fibre phenotype may influence long-term metabolic outcomes. Evidence from epidemiological studies, experimental models, and mechanistic research suggests that maternal metabolic disturbances, including hyperglycaemia, obesity, and systemic inflammation, can influence foetal development through metabolic and inflammatory pathways affecting both neural and muscular components of the motor system. These findings support the hypothesis that the motor neuromuscular axis may represent a structural interface linking early developmental exposures to long-term metabolic regulation and risk of metabolic syndrome.

## 1. Introduction

Over the past decades, it has become widely recognised that health and disease risk across the lifespan are influenced by environmental exposures occurring during critical periods of development. This concept is formalised within the framework of the Developmental Origins of Health and Disease (DOHaD), which proposes that environmental stimuli during gestation and early life can induce long-lasting physiological adaptations capable of modifying susceptibility to disease in adulthood [1,2,3,4,5]. Evidence derived from epidemiological, experimental and clinical studies indicates that alterations in the intrauterine environment, including nutritional restriction or excess, maternal inflammation and gestational hyperglycaemia, can permanently programme multiple physiological systems involved in the regulation of energy metabolism [6,7].

A substantial proportion of the evidence accumulated in this field has focused on tissues traditionally associated with metabolic homeostasis. Alterations in the development of the liver, endocrine pancreas and adipose tissue have been extensively documented in studies of metabolic programming. Adverse nutritional exposures during gestation may modify the structural and functional organisation of the liver, influencing processes such as lipogenesis, substrate oxidation and hepatic glucose production [8,9,10]. Similarly, disturbances in pancreatic development may impair the formation and function of pancreatic β-cells, affecting insulin secretion and glycaemic regulation. Changes in adipose tissue expansion and function have also been associated with future metabolic disturbances, including obesity, insulin resistance and metabolic syndrome [6,11,12,13,14,15,16].

Despite these advances, the predominant focus on classical metabolic organs may limit a comprehensive understanding of the mechanisms linking early-life exposures to metabolic disease risk. Systemic energy metabolism emerges from coordinated interactions among multiple physiological systems, including the nervous, musculoskeletal and endocrine systems [17,18,19,20]. Nevertheless, most investigations of metabolic programming still devote comparatively limited attention to the development of the motor system and to the organisation of the neuromuscular axis. Considering that skeletal muscle represents the primary site of insulin-stimulated peripheral glucose uptake and plays a central role in the regulation of metabolic flexibility, the integration between neural control and muscle metabolism may constitute an important component in shaping metabolic trajectories across the lifespan [21,22,23,24,25].

The development of the neuromuscular system involves highly coordinated processes, including motor neuron differentiation, axonal growth, formation of the neuromuscular junction and metabolic specialisation of muscle fibres. These events occur during critical windows of embryonic and postnatal development, during which genetic factors, neural activity and metabolic signals interact to define the functional organisation of motor units [26,27,28,29,30]. Alterations in the metabolic environment during these periods may potentially influence the maturation of the motor system and consequently modify metabolic properties of skeletal muscle, including oxidative capacity, fibre composition and insulin sensitivity [31,32,33,34,35].

In this context, the present review advances the hypothesis that the motor neuromuscular axis represents an underexplored structural dimension of metabolic programming. More specifically, we propose that early-life metabolic exposures may influence neuromuscular development and, through alterations in motor unit organisation and skeletal muscle metabolic phenotype, contribute to distinct metabolic trajectories throughout life.

Accordingly, this review aims to integrate evidence from developmental biology, neuroscience and metabolic physiology to discuss the potential role of the motor neuromuscular axis in metabolic programming. By examining mechanisms of neuromuscular development, interactions between neural activity and muscle metabolism, and their possible implications for metabolic disease, we seek to expand the conceptual framework of the DOHaD paradigm and highlight new directions for experimental and translational research.

## 2. DOHaD and Metabolic Programming: Current State of Knowledge

The high global prevalence of non-communicable diseases (NCDs) has been increasingly associated with early-life environmental exposures, highlighting the importance of the gestational and perinatal periods in shaping health across the lifespan [36,37]. Evidence indicates that the intrauterine environment and the earliest stages of postnatal development significantly influence the physiological trajectory of the offspring, modulating the risk of disease and morphological alterations that may manifest later in life [38,39]. Understanding these mechanisms of biological plasticity is essential for explaining complex clinical outcomes and for informing preventive strategies aimed at improving maternal and foetal health [12,40,41].

Within this context, the concept of foetal programming emerged, proposing that environmental stimuli during critical windows of development can induce long-lasting adaptations in the organism [2,3,41,42,43]. As scientific evidence accumulated, this concept evolved into the broader paradigm of the Developmental Origins of Health and Disease (DOHaD), which integrates both intrauterine exposures and postnatal environmental influences as determinants of disease risk across the life course [41].

### 2.1. Epidemiological and Experimental Foundations of DOHaD

The epidemiological foundations of foetal programming can be traced back to the early twentieth century, when population studies suggested that socioeconomic and sanitary conditions during childhood were associated with mortality risk in adulthood [1,44]. However, the hypothesis that the gestational environment could influence the risk of chronic diseases gained greater prominence during the 1970s and 1980s, when epidemiological studies began to demonstrate associations between maternal nutritional restriction during pregnancy and increased incidence of cardiovascular and metabolic diseases later in life [1,45,46,47,48].

David Barker played a pivotal role in consolidating this field by demonstrating, through analyses of historical records from England and Wales, that regions with higher infant mortality rates in the early twentieth century also exhibited higher mortality from cardiovascular diseases decades later [1,45,46,47,48]. These findings led to the formulation of the foetal origins of adult disease hypothesis, which proposes that foetal growth and intrauterine conditions may influence the risk of chronic disease throughout life [49,50,51].

Additional evidence was provided by historical studies involving individuals exposed to the Dutch Hunger Winter of 1944–1945. Exposure to famine during foetal development was associated with an increased risk of obesity, diabetes and cardiovascular disease in adulthood [2,45]. These observations contributed to the formulation of the thrifty phenotype hypothesis, which proposes that metabolic adaptations developed in response to prenatal undernutrition may become maladaptive when individuals are later exposed to environments of nutritional abundance [5,6,52]. This concept was later integrated into the developmental mismatch paradigm, which describes the discrepancy between the environment anticipated during foetal development and the environment encountered after birth [53,54].

### 2.2. Structural, Functional and Epigenetic Programming

The DOHaD paradigm is based on the high degree of biological plasticity observed during development, which allows organisms to undergo phenotypic adaptations in response to environmental cues during critical developmental periods [53,54]. These adaptations may involve structural modifications in organs, functional changes in physiological systems and long-lasting alterations in gene expression.

Among the biological mechanisms proposed to mediate these processes are alterations in signalling pathways sensitive to metabolic and hormonal states, including mTOR, AMPK, IGF-1/insulin signalling and glucocorticoid receptors. These pathways regulate fundamental processes such as cellular proliferation, tissue differentiation and apoptosis, thereby influencing the structural development of organs and tissues [36,55,56].

In gestational diabetes, for example, hyperglycaemia may promote the formation of advanced glycation end-products (AGEs), which interact with their receptors (RAGE) and activate signalling cascades associated with oxidative stress, inflammation and apoptosis [55]. These processes can affect the development of several metabolically relevant organs, including the pancreas, liver and kidneys, thereby increasing susceptibility to metabolic disorders in later life.

In addition to metabolic and hormonal disturbances, epigenetic mechanisms have been widely implicated in foetal programming. Epigenetics refers to heritable modifications in gene expression that occur without changes in the nucleotide sequence of DNA [57,58]. The principal mechanisms include DNA methylation, post-translational histone modifications and regulation by non-coding RNAs [56,59]. These modifications can lead to persistent alterations in gene expression and cellular function, contributing to the maintenance of metabolically programmed phenotypes throughout life. For instance, intrauterine growth restriction has been associated with epigenetic alterations in genes involved in energy metabolism, including IGF2 and CPT1A [60].

### 2.3. Impact of the Maternal Metabolic Environment

Several factors present during pregnancy can influence foetal metabolic programming, including nutritional restriction or excess, maternal hyperglycaemia, systemic inflammation, stress, glucocorticoid exposure and placental dysfunction [61]. Maternal undernutrition may impair the development of metabolically active organs such as the endocrine pancreas, resulting in a reduced number of pancreatic β-cells. In contrast, maternal obesity may promote foetal hyperinsulinaemia and alterations in insulin sensitivity, thereby increasing the risk of metabolic syndrome in later life [55,60].

Furthermore, maternal inflammation associated with obesity or metabolic syndrome may alter the intrauterine environment through the release of pro-inflammatory cytokines, hormonal changes and modifications in nutrient availability. These factors may interfere with tissue development and affect the structural and functional organisation of multiple physiological systems [7,62,63,64].

### 2.4. Limitations of Models Centred Exclusively on Classical Metabolic Tissues

A substantial proportion of research on metabolic programming has focused on tissues traditionally associated with energy homeostasis, including the liver, pancreas and adipose tissue. These organs play central roles in the regulation of glucose and lipid metabolism and have therefore been historically prioritised in DOHaD research [43,47,65,66]. However, increasing evidence indicates that metabolic programming involves complex interactions among multiple physiological systems. Organs and tissues not traditionally classified as metabolic, such as the nervous system and the musculoskeletal system, also contribute significantly to the regulation of systemic energy homeostasis [67,68,69,70].

Consequently, models centred exclusively on classical metabolic tissues may limit a comprehensive understanding of the mechanisms linking early-life environmental exposures to metabolic disease risk in adulthood. Integrative approaches that incorporate interactions among different physiological systems may therefore expand current knowledge of the biological bases of metabolic programming and reveal additional mechanisms involved in metabolic regulation across the lifespan [71,72,73].

## 3. Development of the Motor Neuromuscular Axis During Critical Windows

**Figure 1:**
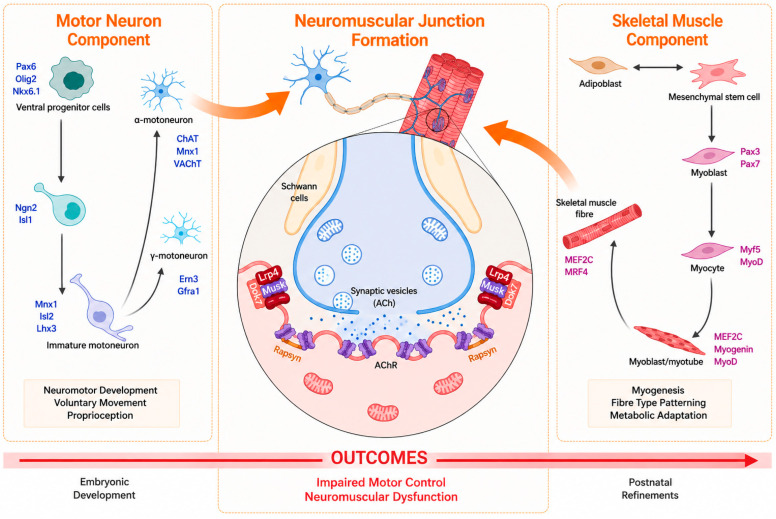
Schematic representation of neuromuscular junction (NMJ) formation and maturation, integrating motor neuron and skeletal muscle components throughout development. On the left, the differentiation stages of motor neurons from ventral progenitor cells are illustrated, highlighting transcription factors involved in neuronal specification and the formation of α- and γ-motoneurons. On the right, myogenesis is depicted from mesenchymal stem cells and myoblasts to mature skeletal muscle fibres, including the major myogenic regulatory factors involved in muscle development. In the centre, the structural organisation of the NMJ is shown, including the presynaptic terminal, Schwann cells, synaptic vesicles containing acetylcholine (ACh), and postsynaptic components such as acetylcholine receptors (AChRs), MuSK, Lrp4, Dok7, and rapsyn, which are essential for synaptic stabilisation and functionality. The coordinated development of neuronal and muscular components is critical for motor control, voluntary movement, proprioception, muscle fibre patterning, and metabolic adaptation. Disruptions in these processes may lead to impaired motor control and neuromuscular dysfunction.

The development of the motor neuromuscular axis represents a highly coordinated process involving the functional integration of motor neurons, peripheral nerves, neuromuscular junctions and skeletal muscle fibres (Figure 1). Motor neuron development is initiated by neuromesodermal progenitors located in the caudal embryo, whose maintenance and differentiation are regulated by gradients of Wnt, fibroblast growth factor (FGF) and retinoic acid signalling, providing temporal and spatial control of neurogenesis along the rostrocaudal axis [74,75]. This system does not merely execute motor commands; rather, it constitutes a complex functional axis whose structural and physiological organisation is established during critical developmental periods. During these temporal windows, processes such as neuronal differentiation, axonal growth, synapse formation and metabolic specialisation of muscle fibres occur in an integrated manner and are highly sensitive to molecular and environmental signals. Within the framework of DOHaD, it has been proposed that alterations in the intrauterine or neonatal metabolic environment may influence the maturation of this system, modulating functional properties of skeletal muscle and motor control across the lifespan [76,77,78,79].

### 3.1. Motor Neuron Differentiation and Organisation of Motor Pools

The functional diversity of the motor system originates during embryonic development of the spinal cord, when populations of motor neurons are generated from progenitors located in the ventral region of the neural tube. This process involves the sequential activation of genetic programmes regulated by gradients of morphogens and transcription factors that determine cellular identity and the final positioning of motor neurons along the spinal axis. In addition, this process is coordinated by dynamic interactions between morphogen gradients and transcriptional networks, including cross-repressive mechanisms that refine progenitor domain identity [80]. As these neurons differentiate, their axons extend towards peripheral muscles, establishing specific connections that form the functional basis of motor units [81,82,83,84,85]. During this developmental phase, an initial overproduction of motor neurons is followed by programmed cell death, with neuronal survival depending on competition for limiting target-derived trophic factors [86].

#### 3.1.1. Spinal Patterning

The dorsoventral patterning of the spinal cord is regulated by extrinsic signals, among which the morphogen Sonic Hedgehog (SHH) plays a central role. Secreted by the notochord and the floor plate, SHH establishes a concentration gradient that instructs ventral progenitor cells to acquire distinct cellular identities. Among the resulting domains is the pMN domain, characterised by the expression of transcription factors such as Olig2, Nkx6.1 and Pax6, which are responsible for the generation of spinal motor neurons [87,88]. In this context, neuronal specification occurs through activation of lineage-specific transcriptional programmes and repression of alternative cell fates, with Olig2 promoting cell cycle exit through inhibition of Notch signalling and activation of proneural factors [80,89].

Following cell cycle exit, these progenitors activate transcriptional programmes that determine the organisation of motor neurons into longitudinal columns along the spinal cord. Among these structures are the medial motor column (MMC), which innervates axial musculature, and the lateral motor column (LMC), responsible for the innervation of limb muscles. Within these columns, neurons are organised into motor pools, each containing all motor neurons responsible for innervating a specific muscle. The functional identity of these pools is regulated by combinatorial codes of Hox family transcription factors, which control axonal growth and the selection of peripheral muscle targets [90,91,92,93]. These identity programmes are further stabilised by epigenetic mechanisms that maintain motor neuron subtype specification and somatotopic organisation [91,94,95].

In addition to their organisation into motor pools, the motor system exhibits functional diversity associated with distinct motor neuron subtypes. Alpha motor neurons innervate extrafusal muscle fibres responsible for force generation, whereas gamma motor neurons innervate intrafusal fibres within muscle spindles and participate in the regulation of proprioception. Beta motor neurons possess the capacity to innervate both fibre types. Within the population of alpha motor neurons, further subtypes are associated with the functional properties of motor units, including slow (S), fast fatigue-resistant (FR) and fast fatigable (FF) units, which differ in cell size, recruitment threshold and metabolic characteristics [96,97,98,99]. The survival and maturation of these neurons depend on trophic signalling mediated by receptors such as Trk and p75NTR, as well as GDNF signalling via the GFRα1–RET complex, which activates intracellular pathways including PI3K/Akt and MAPK [100,101].

#### 3.1.2. Synaptic Competition and Activity-Dependent Refinement

Following axonal growth and initial contact with muscle fibres, a period of synaptic refinement begins that is fundamental for the final organisation of the motor unit. During early postnatal development, a single muscle fibre may receive innervation from multiple axons originating from different motor neurons, a phenomenon known as polyinnervation. As development progresses, a process of competitive synaptic elimination occurs, whereby only a single functional connection is maintained at each muscle fibre [102,103,104,105,106].

This refinement depends strongly on neural activity. Differences in firing patterns among competing axons influence the stability of synaptic connections, favouring the maintenance of more active terminals and the withdrawal of less efficient ones. This process involves intracellular signalling pathways with antagonistic roles, in which protein kinase A (PKA) delays axonal elimination whereas protein kinase C (PKC) promotes synapse loss, and is dependent on calcium influx through voltage-gated channels, particularly of the L and P/Q types [107,108,109]. Skeletal muscle plays an active role in this process by releasing trophic factors that regulate the survival and stability of neuromuscular connections. Among these factors, IGF-1 and GDNF act as retrograde signals that promote the stabilisation of functionally active synaptic terminals [110,111]. In addition, terminal Schwann cells participate in synaptic remodelling by interacting with the motor endplate and facilitating structural turnover during axonal competition [112,113].

Competitive synaptic elimination is essential for the establishment of functional motor units and for the precision of motor control. Disruptions during this critical developmental window may lead to abnormal motor unit organisation or reduced efficiency of neuromuscular transmission [103,104,111,114].

#### 3.1.3. Sensitivity to Metabolic and Inflammatory Environments

The development of the motor system occurs within a tightly regulated metabolic environment. In addition to supporting cellular processes, metabolism actively regulates neuronal differentiation through metabolic reprogramming characterised by increased oxidative phosphorylation, mitochondrial remodelling and integration of glycolytic and glutamine pathways [115,116,117]. However, metabolic disturbances associated with conditions such as gestational diabetes, maternal obesity or metabolic syndrome may significantly alter the intrauterine environment, exposing the foetus to elevated levels of glucose, free fatty acids and pro-inflammatory cytokines [32,33,118,119,120].

These metabolic factors may directly influence cellular processes essential for neural development, including neuronal proliferation, axonal growth and synapse formation. Saturated fatty acids, for example, can activate inflammatory pathways through Toll-like receptors, particularly TLR4, which are expressed in neurons and glial cells. Activation of these pathways may induce neuroinflammatory processes and oxidative stress capable of affecting the survival and function of developing motor neurons [121,122].

Furthermore, elevated glucose levels may increase the production of reactive oxygen species and impair mitochondrial function, processes that can interfere with axonal transport and the formation of functional synaptic connections. Because motor neurons exhibit high energetic demands, alterations in cellular metabolism during critical developmental phases may affect the stability of neuromuscular connections and the final organisation of motor units [123,124,125,126].

### 3.2. Formation and Maturation of the Neuromuscular Junction

The neuromuscular junction represents the specialised synapse responsible for converting the motor neuron action potential into muscle contraction. The formation of this structure involves a highly coordinated sequence of molecular events that regulate the positioning of the nerve terminal, the organisation of the postsynaptic membrane and the stabilisation of neuromuscular connections [29,106,110,127,128]. This pre-patterning reflects the active role of skeletal muscle in establishing a permissive domain for synapse formation [129,130,131].

#### Establishment of the Motor Endplate

Even before the arrival of the motor axon, acetylcholine receptors (AChRs) begin to cluster in the central region of the muscle fibre in a process known as pre-patterning. This mechanism depends on the activity of muscle-specific kinase (MuSK) and the anchoring protein rapsyn, which participate in the initial organisation of receptor clusters within the muscle membrane [70,111,132,133,134].

With the arrival of the nerve terminal, the signalling molecule agrin is released into the synaptic cleft. Agrin binds to the receptor LRP4 on the muscle fibre membrane and activates the MuSK signalling pathway. Activation of this cascade promotes the recruitment of the adaptor protein Dok-7, triggering phosphorylation processes that stabilise clusters of acetylcholine receptors directly beneath the nerve terminal. At the same time, acetylcholine released by the nerve acts as a negative signal that disperses receptor clusters located outside the synaptic region, thereby ensuring the spatial specificity of the motor endplate [132,133,134,135,136]. In parallel, retrograde signals derived from muscle fibres contribute to presynaptic differentiation and alignment of active zones with postsynaptic receptor clusters [137].

During postnatal maturation, the neuromuscular junction undergoes significant morphological transformations, evolving from a relatively simple structure into the complex organisation characteristic of adult muscle. This process includes the formation of deep postsynaptic folds that increase the contact surface between nerve and muscle, as well as the replacement of the foetal gamma subunit of the acetylcholine receptor with the adult epsilon subunit, a transition that modifies the electrophysiological properties of the synapse [110,111,127,128,138,139]. In addition to structural changes, synaptic maturation involves functional plasticity, including adjustments in neurotransmitter release probability, vesicle availability and metabolic sensing pathways such as AMPK, which link cellular energy status to synaptic stability [140,141].

### 3.3. Specification of Muscle Fibre Types and Motor Unit Identity

The specification of skeletal muscle fibre types represents a critical component of the motor neuromuscular axis, emerging from the integration of neural activity and metabolic signalling pathways. Muscle fibres are classified based on myosin heavy chain isoforms into slow and fast types; however, this classification reflects a phenotypic continuum rather than discrete categories [142,143].

Transcriptional coactivators such as PGC-1α play a central role in promoting oxidative fibre phenotypes, whereas calcium-dependent signalling pathways, including calcineurin–NFAT, regulate activity-dependent gene expression [144,145]. Additionally, mitochondrial dynamics and signalling pathways contribute to fibre specification, linking metabolic state to contractile identity [146].

Patterns of motor neuron firing further modulate fibre identity, with tonic low-frequency activity favouring oxidative phenotypes and phasic high-frequency activity promoting glycolytic characteristics [147]. These differences are also reflected in synaptic properties and calcium dynamics, contributing to functional diversity among motor units.

Importantly, early-life metabolic conditions can programme long-term alterations in muscle fibre composition and metabolic flexibility, without necessarily changing fibre number, reinforcing the concept that developmental environments shape the functional properties of the neuromuscular system across the lifespan [148,149,150].

### 3.4. Development of Muscle Proprioceptors: Muscle Spindle and Tendon Organ

The development of muscle proprioceptors occurs in coordination with the assembly of the motor neuromuscular axis, involving the specification and integration of muscle spindle and tendon organ receptors. Muscle spindle formation is initiated by the differentiation of intrafusal muscle fibres, a process induced by sensory-afferent-derived signals that activate specific transcriptional programmes required for intrafusal identity and structural organisation. Primary sensory innervation precedes and instructs spindle morphogenesis, promoting the formation of specialised sensory endings and the establishment of a central non-contractile domain. This process is critically dependent on neurotrophin signalling, particularly NT-3–TrkC pathways, as well as transcriptional regulators such as Egr3, which are required for intrafusal fibre differentiation and maintenance. Subsequent innervation by γ-motor neurons contributes to functional maturation and stabilisation of the receptor, in association with activity-dependent mechanisms during postnatal development [151,152,153,154,155].

The tendon organ develops at the myotendinous interface through the interaction between sensory afferents and the extracellular matrix of the tendon. Its formation depends on the differentiation of sensory endings associated with collagen fibres and on the structural organisation of the myotendinous junction. Mechanotransduction and trophic signalling pathways regulate this process, linking extracellular matrix composition and mechanical load to receptor maturation and functional specification [152,153,156,157].

The maturation of these proprioceptive systems occurs within critical developmental windows and depends on coordinated interactions between sensory input, motor output and muscle differentiation. Disruptions in metabolic or inflammatory environments during these periods may interfere with neurotrophin signalling, transcriptional regulation and mechanosensitive pathways, potentially impairing proprioceptor development and contributing to long-term alterations in sensorimotor integration and neuromuscular function [151,152,155,156,157].

## 4. Maternal Metabolic Environment as a Modulator of Motor Neurodevelopment

**Figure 2:**
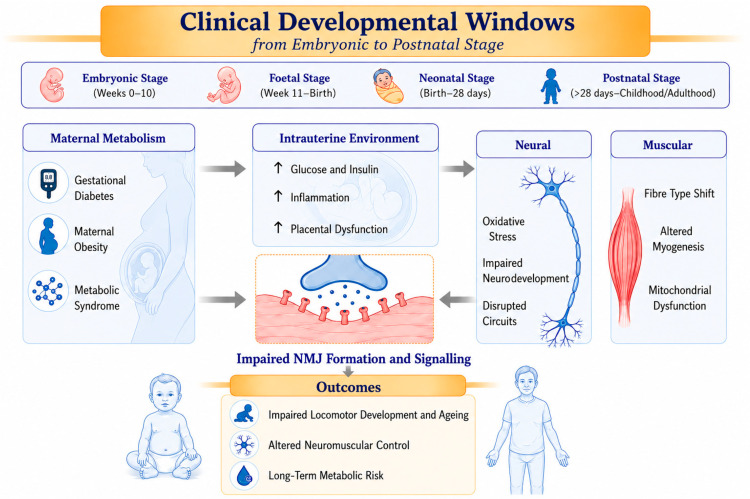
Schematic overview of the critical developmental windows linking maternal metabolic disorders to impaired neuromuscular junction (NMJ) formation and long-term neuromuscular dysfunction, spanning embryonic, foetal, neonatal and postnatal stages. Maternal conditions such as gestational diabetes, maternal obesity and metabolic syndrome contribute to alterations in the intrauterine environment, including increased glucose and insulin levels, inflammation and placental dysfunction. These disturbances may adversely affect both neural and muscular developmental pathways. Neural alterations include oxidative stress, impaired neurodevelopment and disrupted neuronal circuitry, whereas muscular impairments involve fibre type shifts, altered myogenesis and mitochondrial dysfunction. Together, these changes compromise NMJ formation and signalling, potentially leading to impaired locomotor development, altered neuromuscular control, accelerated functional decline during ageing and increased long-term metabolic risk across the lifespan.

The maternal metabolic environment during pregnancy exerts a significant influence on foetal development. Metabolic disturbances associated with conditions such as gestational diabetes, maternal obesity and metabolic syndrome may modify the intrauterine environment through hormonal, inflammatory and nutritional alterations. These changes affect multiple developing systems, including the nervous system and skeletal muscle (Figure 2). Within the DOHaD framework, adverse metabolic exposures during critical periods of gestation have been proposed to influence developmental trajectories of several physiological systems, with long-term consequences for metabolic and neuromuscular regulation across the lifespan [32,158,159,160,161].

Although much of the research in this field has focused on metabolic, cardiovascular or cognitive outcomes, increasing evidence suggests that the motor system may also be sensitive to alterations in the intrauterine metabolic environment. Neuromuscular development involves highly coordinated processes, including motor neuron differentiation, axonal growth, formation of the neuromuscular junction and maturation of muscle fibres. These events occur during critical windows of embryonic and foetal development, during which metabolic or inflammatory disturbances may interfere with the structural and functional organisation of this system [110,162,163,164]. Importantly, much of the available evidence derives from studies of the developing brain, particularly the hypothalamic and cortical regions. Therefore, when discussing the motor neuromuscular axis, the following sections distinguish between direct evidence and biologically plausible inferences based on related neuronal populations.

### 4.1. Effects of Gestational Hyperglycaemia on Neural Development

Gestational hyperglycaemia represents one of the most extensively studied metabolic disturbances in the context of foetal development. During pregnancy, elevated maternal glucose levels can cross the placenta and directly modify the foetal metabolic environment. Prolonged exposure to hyperglycaemia may influence fundamental cellular processes in the developing nervous system, including neuronal proliferation, differentiation and neural circuit formation [23,165,166,167].

Clinical and experimental studies have demonstrated that gestational diabetes is associated with alterations in central nervous system development in the offspring, including modifications in brain structure and neurocognitive function. Although the precise mechanisms remain incompletely understood, foetal hyperglycaemia is believed to induce oxidative stress, disturbances in cellular energy metabolism and alterations in insulin signalling within developing neural tissue. These processes may interfere with neuronal maturation and the organisation of neural circuits responsible for motor control [168,169,170,171].

In addition, metabolic disturbances associated with gestational diabetes, such as foetal hyperinsulinaemia and alterations in lipid metabolism, may modify the intrauterine hormonal environment. These factors have the potential to influence the expression of genes involved in neural development, synaptic connectivity and the maturation of motor circuits. Although most evidence relates to cognitive development and central nervous system organisation, similar mechanisms may also affect neural circuits involved in motor control [169,170,171,172,173]. Although direct evidence in motor neurons remains limited, similar mechanisms may also affect neural circuits involved in motor control by analogy with observations in other neuronal populations.

### 4.2. Inflammation Associated with Maternal Obesity and Potential Impacts on Neuronal Differentiation

Maternal obesity is frequently associated with a state of chronic low-grade metabolic inflammation, characterised by increased circulating levels of pro-inflammatory cytokines, adipokines and inflammatory mediators. During pregnancy, these mediators may cross the placenta or influence placental function, altering the intrauterine environment and potentially interfering with foetal development [174,175,176,177,178].

Inflammatory cytokines such as TNF-α and IL-6 have been implicated in the modulation of neuronal differentiation processes and synaptic plasticity. Experimental studies indicate that exposure to inflammatory environments during critical periods of neural development may alter neural progenitor proliferation, influence cellular migration and modify the organisation of neuronal circuits [179,180,181].

Beyond direct effects on the nervous system, maternal systemic inflammation may also alter placental function, modifying the transport of nutrients, hormones and growth factors to the foetus. Changes in this exchange system may affect tissues highly dependent on energy availability and metabolic signalling, such as the nervous system and skeletal muscle. Although the effects of maternal inflammation on neuromuscular development remain incompletely characterised, the available evidence supports a broader influence of inflammatory intrauterine environments on neural circuit development, with motor pathways representing a biologically plausible extension of these observations [179,182,183,184].

### 4.3. Experimental Evidence from Animal Models

Animal models have been widely employed to investigate the effects of the maternal metabolic environment on foetal development. Studies using models of maternal obesity, high-fat diets or experimental gestational diabetes demonstrate that metabolic disturbances during pregnancy may produce long-lasting effects on the metabolism, body composition and neural function of the offspring [185,186,187,188,189,190].

In several experimental models, maternal high-fat diets have been associated with alterations in brain inflammation, neurotransmitter signalling and neural circuit development in offspring. These alterations include changes in the expression of genes involved in neuronal plasticity, energy metabolism and inflammatory pathways within the central nervous system [191,192,193,194,195,196].

Furthermore, animal studies indicate that adverse metabolic exposures during gestation may influence the development of regulatory systems involved in appetite control, energy metabolism and motor behaviour. Most experimental studies have focused on hypothalamic and other metabolically relevant brain regions. Nevertheless, the underlying mechanisms, including neuroinflammation, altered energy sensing and impaired neuronal maturation, are fundamental developmental processes that are equally relevant to motor circuit formation [191,192,193,197,198,199].

Direct evidence supporting the involvement of the motor neuromuscular axis in developmental programming has begun to emerge from experimental models of maternal nutritional imbalance. Maternal protein restriction during pregnancy and lactation has been shown to alter neuromuscular junction development, reducing NMJ number and inducing structural abnormalities in offspring skeletal muscles, including changes in synaptic folding and acetylcholine receptor subunit expression [200,201,202,203,204]. These findings indicate that adverse maternal nutritional environments may directly influence neuromuscular connectivity during critical developmental periods, rather than affecting metabolic outcomes exclusively through classical metabolic organs.

Additional evidence suggests that early nutritional disturbances may also influence the metabolic phenotype of skeletal muscle. Maternal protein restriction has been associated with persistent alterations in muscle fibre development, favouring glycolytic characteristics and modifying the expression of genes involved in oxidative metabolism and metabolic flexibility, including PGC-1α, CPT1a, UCP3 and PPARα [149,150,205,206]. These observations support the hypothesis that developmental programming may affect not only muscle mass and structure but also the long-term metabolic properties of skeletal muscle.

### 4.4. Indirect Evidence Involving Myogenesis, Fibre Type Patterning and Muscle Organisation

Although direct evidence linking the maternal metabolic environment to the organisation of the neuromuscular system remains limited, numerous studies indicate that early metabolic exposures can influence skeletal muscle development. Alterations in foetal myogenesis, muscle fibre composition and muscle metabolic capacity have been observed in experimental models of maternal obesity and gestational diabetes [207,208,209].

These alterations include changes in the proportion of oxidative and glycolytic muscle fibres, mitochondrial density and the capacity for substrate oxidation. Because the functional organisation of skeletal muscle is closely dependent on the interaction between muscle fibres and motor neurons, structural or metabolic changes in muscle tissue may reflect adaptations within the neuromuscular axis [114,210,211].

Moreover, skeletal muscle development and neuromuscular junction formation occur in a coordinated manner during embryonic and foetal development. Alterations in the metabolic environment may influence growth factors, hormonal signals and energy availability required for these processes, potentially affecting the structural organisation of the neuromuscular system [138,140,212].

### 4.5. Convergence Between Metabolic Environment and Neuromuscular Maturation

The processes regulating neuromuscular development involve complex interactions between genetic signals, neural activity and metabolic factors. During embryonic and foetal development, motor neuron differentiation, axonal growth and neuromuscular junction formation occur in close coordination with skeletal muscle development [109,110,213,214].

Alterations in the maternal metabolic environment may influence these processes through multiple mechanisms, including changes in nutrient availability, hormonal signalling, systemic inflammation and cellular energy metabolism. These factors may affect both neural and muscular development, suggesting potential points of convergence between metabolic environment and neuromuscular maturation [200,201,215,216].

Within the context of metabolic programming, these interactions suggest that the development of the neuromuscular axis may represent a relevant component linking early metabolic exposures to the risk of metabolic diseases later in life. The available findings support the hypothesis that the intrauterine metabolic environment contributes to the structural and functional organisation of the neuromuscular system. This framework integrates direct evidence from neuromuscular studies with complementary developmental evidence from related neuronal systems, providing a coherent biological model for the role of the motor neuromuscular axis in metabolic programming.

## 5. Activity-Dependent Programming and Metabolic Trajectories Across the Lifespan

The development of the neuromuscular system is regulated by a complex interaction between intrinsic genetic programmes and activity-dependent signals. During embryonic development and the early stages of postnatal life, activity-dependent mechanisms contribute to the establishment of neuromuscular organisation and function. As discussed in Section 3, these developmental processes occur during critical windows of plasticity and may influence long-term physiological characteristics. In this context, alterations in neural activity during development may contribute to persistent changes in metabolic regulation across the lifespan [217,218,219].

**Figure 3:**
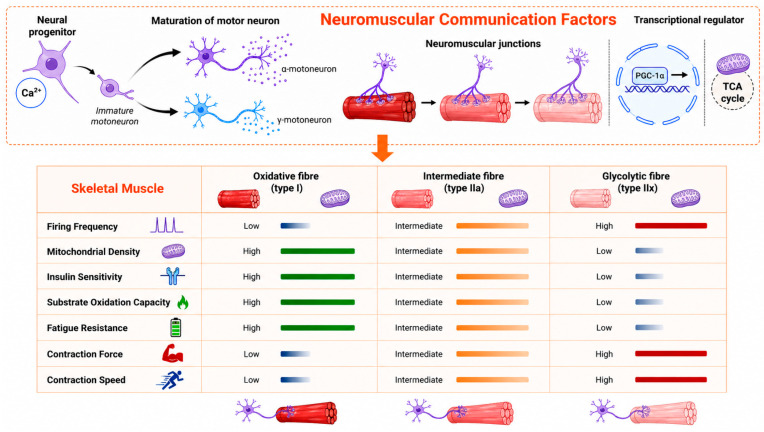
Overview of neuromuscular communication factors associated with motor neuron maturation, neuromuscular junction (NMJ) formation, and skeletal muscle fibre specialisation. The upper panel illustrates the progression from neural progenitor cells to immature and mature α- and γ-motoneurons, the establishment of NMJs, and transcriptional regulation involving PGC-1α and mitochondrial metabolic pathways, including the tricarboxylic acid (TCA) cycle. The lower panel summarises the physiological and metabolic characteristics of skeletal muscle fibre types. Oxidative fibres (type I) are characterised by low firing frequency, high mitochondrial density, elevated insulin sensitivity, increased substrate oxidation capacity, high fatigue resistance, and slower contraction force and speed. Intermediate fibres (type IIa) display mixed metabolic and functional properties, whereas glycolytic fibres (type IIb) exhibit high firing frequency, reduced mitochondrial density, lower insulin sensitivity, diminished oxidative capacity, reduced fatigue resistance, and faster, more forceful contractions. These neuromuscular and metabolic adaptations are critical determinants of muscle performance, energy metabolism, and functional plasticity.

Beyond its role in the structural organisation of the motor system, neural activity also exerts a direct influence on skeletal muscle metabolism (Figure 3). Activity-dependent signalling regulates substrate utilisation, mitochondrial function and oxidative capacity, thereby contributing to the establishment of physiological characteristics that may persist throughout life and influence metabolic performance. These adaptations are mediated by molecular pathways involved in neuromuscular and metabolic regulation, as discussed in Section 3.

### 5.1. Role of Spontaneous Electrical Activity During Development

During the early stages of nervous system development, patterns of spontaneous electrical activity play an essential role in the formation and refinement of neural circuits. Even before the complete maturation of sensory and motor pathways, developing neurons exhibit spontaneous firing patterns that contribute to the functional organisation of neuronal networks [220].

Within the motor system, this early electrical activity participates in the guidance of axonal growth, the formation of synaptic connections and the stabilisation of neuromus-cular junctions. Experimental studies demonstrate that alterations in neural activity during development may affect both the formation of neuromuscular synapses and the organisation of motor units [221]. Activity-dependent calcium signalling acts as a central mediator of these processes by regulating transcriptional programmes associated with neuronal differentiation, survival and synaptic plasticity [221].

During the early postnatal period, neuromuscular connections undergo an activity-dependent refinement process. Initially, each muscle fibre may receive innervation from multiple motor axons; however, as development progresses, a process of competitive synaptic elimination occurs, whereby only a single functional connection is retained. This refinement depends on electrical activity and on interactions among the motor neuron, the muscle fibre and supporting cells present at the neuromuscular junction, contributing to the establishment of the functional organisation of motor units. Disruptions in electrical activity during critical developmental periods, including those associated with hypoxia, inflammation or metabolic imbalance, may lead to persistent alterations in neuromuscular organisation and function [105,106,222].

### 5.2. Relationship Between Neural Firing Patterns and Muscle Oxidative Capacity

Skeletal muscle displays considerable functional diversity and is composed of muscle fibres with distinct contractile and metabolic properties. This diversity is closely associated with the activation patterns of the motor units that innervate these fibres. Motor units characterised by low firing frequency and sustained activation tend to innervate oxidative muscle fibres, which exhibit higher mitochondrial density, greater substrate oxidation capacity and increased resistance to fatigue. In contrast, motor units recruited during conditions requiring greater force production typically innervate fast-contracting glycolytic muscle fibres with lower oxidative capacity [223].

Classic neural stimulation experiments have demonstrated that patterns of electrical activation can directly influence the metabolic phenotype of skeletal muscle. Experimental manipulation of motor nerve stimulation frequency is capable of inducing changes in both the contractile and metabolic properties of muscle fibres, including alterations in oxidative enzyme activity, mitochondrial density and the expression of contractile protein isoforms [224,225]. These adaptations are mediated by activity-dependent signalling pathways regulating mitochondrial biogenesis and oxidative metabolism, as discussed in Section 3.

These findings indicate that the motor nervous system plays an important role in regulating the metabolic specialisation of skeletal muscle. The firing pattern of motor neurons acts as a physiological signal capable of modulating the metabolic identity of muscle fibres, thereby establishing a functional link between neural control and energy metabolism. Although skeletal muscle retains plasticity throughout life, this capacity is particularly pronounced during early developmental stages, suggesting that early-life neuromuscular activity may exert long-lasting effects on muscle metabolic properties [217].

### 5.3. Metabolic Flexibility and Insulin Sensitivity

Skeletal muscle represents one of the principal tissues responsible for insulin-stimulated glucose uptake in the body. The efficiency of this process depends on the ability of muscle tissue to switch between different energy substrates in response to changing physiological conditions, a phenomenon known as metabolic flexibility [226]. Metabolic flexibility refers to the capacity to adjust the utilisation of carbohydrates and lipids according to nutritional state and physical activity levels.

Muscles with greater oxidative capacity typically display higher mitochondrial density, increased activity of oxidative enzymes and greater insulin sensitivity, characteristics that favour efficient utilisation of energy substrates. In individuals with insulin resistance or type 2 diabetes, reductions in muscle oxidative capacity, alterations in fibre type composition and decreased metabolic flexibility are frequently observed [227,228].

In addition to insulin-dependent pathways, muscle contraction can stimulate glucose uptake through insulin-independent mechanisms, highlighting the role of neuromuscular activity in metabolic regulation [229]. These observations suggest that the structural and functional properties of skeletal muscle exert a direct influence on systemic metabolic regulation [230].

### 5.4. Potential Contribution of the Motor Axis to Metabolic Trajectories in Adulthood

Given the strong interaction between neural activity and muscle metabolism, it has been proposed that the neuromuscular axis may play a significant role in shaping metabolic trajectories across the lifespan. The development of the motor system occurs during critical periods of embryonic and postnatal development, during which genetic, environmental and metabolic factors may influence the organisation of motor units and the metabolic specialisation of skeletal muscle [128,231].

Alterations in the metabolic environment during these phases may potentially affect both neural and muscular development, thereby modifying the functional architecture of the neuromuscular system. Because skeletal muscle metabolism depends largely on motor unit activity, changes in motor unit organisation or neural recruitment patterns may have important implications for the metabolic capacity of muscle throughout life [232].

Beyond its contractile function, skeletal muscle also acts as an endocrine organ through the secretion of myokines in response to contractile activity. These signalling molecules can modulate systemic metabolism by influencing glucose uptake, lipid oxidation and energy expenditure [22,233]. In this context, muscle-derived factors have been implicated in processes such as adipose tissue remodelling and systemic metabolic regulation, including the induction of thermogenic programmes in adipose tissue [234].

Although direct evidence remains limited, the integration of knowledge from developmental neurobiology, muscle physiology and metabolic biology suggests that the neuromuscular axis may represent an important component linking early environmental exposures to the risk of metabolic diseases in adulthood [235].

## 6. The Motor Neuromuscular Axis as a Structural Determinant of Metabolic Flexibility

**Figure 4:**
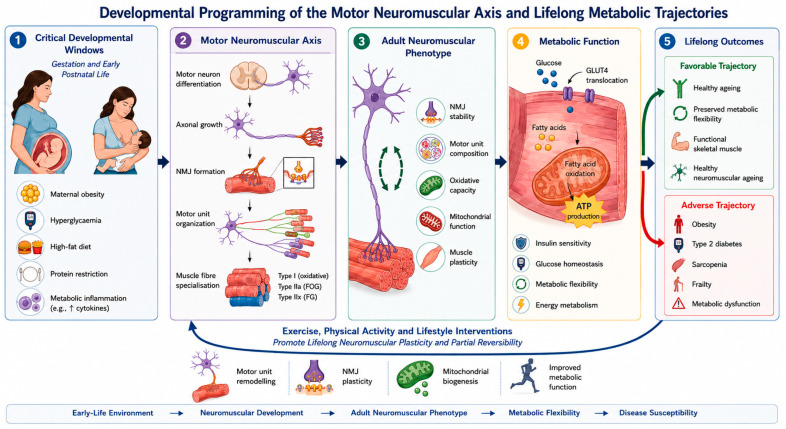
Developmental programming of the motor neuromuscular axis and its potential influence on lifelong metabolic trajectories. Adverse metabolic exposures during critical developmental windows, including gestation and early postnatal life, may influence the development of the motor neuromuscular axis through effects on motor neuron differentiation, neuromuscular junction (NMJ) formation, motor unit organisation and skeletal muscle fibre specialisation. These developmental processes may contribute to the establishment of adult neuromuscular phenotypes characterised by differences in NMJ stability, motor unit composition, mitochondrial function, oxidative capacity and muscle plasticity. Because skeletal muscle is a major site of insulin-stimulated glucose uptake and energy metabolism, alterations in neuromuscular organisation may influence metabolic flexibility, insulin sensitivity and glucose homeostasis. Consequently, developmental perturbations affecting the neuromuscular axis may contribute to differential susceptibility to metabolic disorders, including obesity, type 2 diabetes, sarcopenia and frailty. The feedback loop illustrates the potential for lifelong neuromuscular plasticity, whereby physical activity, exercise and lifestyle interventions may promote motor unit remodelling, NMJ plasticity, mitochondrial biogenesis and improvements in metabolic function, partially attenuating the long-term consequences of early-life programming.

Although the evidence discussed throughout this review suggests that early-life metabolic disturbances may influence multiple components of the neuromuscular system, the mechanisms linking developmental programming to long-term metabolic outcomes remain incompletely understood. The central hypothesis proposed in this review is that the motor neuromuscular axis may function as a biological interface connecting early environmental exposures to lifelong regulation of skeletal muscle metabolism and metabolic flexibility. Because motor neurons, neuromuscular junctions and skeletal muscle fibres develop during critical developmental windows and remain functionally integrated throughout life, alterations occurring during these periods may have persistent consequences for neuromuscular organisation and metabolic health. Figure 4 summarises this conceptual framework, illustrating how adverse metabolic exposures during gestation and early postnatal life may influence neuromuscular development, shape adult neuromuscular phenotypes and ultimately contribute to differential susceptibility to metabolic diseases, while also highlighting the potential modulatory effects of lifelong neuromuscular plasticity and exercise interventions.

### 6.1. Operational Definition of the Motor Neuromuscular Axis

The motor neuromuscular axis can be defined as an integrated functional system comprising the alpha motor neuron located in the anterior horn of the spinal cord, its peripheral axon, the neuromuscular junction and the set of skeletal muscle fibres it innervates, collectively forming the motor unit [140,212,236,237]. Within this system, spinal motor neurons integrate excitatory and inhibitory inputs from multiple neural circuits, converting this synaptic integration into specific patterns of electrical discharge that propagate along motor axons to the neuromuscular junction. At the motor endplate, the release of acetylcholine converts the electrical signal into chemical synaptic transmission, triggering action potentials in the muscle fibre membrane and initiating the process of excitation–contraction coupling [237,238].

In this context, motor neurons not only control muscle contraction but also determine activation patterns that regulate muscle fibre recruitment and the energetic demand associated with contraction. In response to these activation patterns, skeletal muscle exhibits substantial metabolic plasticity, adapting its functional architecture through changes in mitochondrial density, expression of metabolic enzymes and availability of energy substrate transporters [239,240]. At the same time, muscle releases myokines and neurotrophic factors capable of exerting retrograde influences on synaptic stability and maintenance of motor circuits. The motor neuromuscular axis should therefore be understood as a bidirectional system in which neural activity, motor unit organisation and muscle metabolism interact continuously to regulate the efficiency of energy substrate utilisation [238,240].

### 6.2. Integration Between Neural Control, Muscle Phenotype and Glycaemic Homeostasis

Systemic glycaemic homeostasis depends to a large extent on the capacity of skeletal muscle to uptake, oxidise and store glucose in response to hormonal and neural signals. However, this capacity is not determined solely by intrinsic properties of muscle tissue but also by the functional organisation of the motor unit and the neural control patterns regulating its activity [92,236]. The frequency and duration of motor neuron firing determine not only the mechanical recruitment of muscle fibres but also the intensity of energy metabolism associated with contraction, directly influencing substrate flux through metabolic pathways such as cytosolic glycolysis and mitochondrial oxidative phosphorylation [238,241,242].

The motor neuromuscular axis displays considerable physiological plasticity. Through excitation–transcription coupling mechanisms, patterns of neural activity can modulate the expression of proteins associated with oxidative metabolism, mitochondrial biogenesis and substrate transport, including transporters such as GLUT4 and CD36 [238,242]. These adaptations involve activity-dependent signalling pathways and transcriptional regulators associated with mitochondrial biogenesis, substrate utilisation and oxidative metabolism, as discussed in Section 3.

In addition to these pathways, AMP-activated protein kinase (AMPK) functions as a central energy sensor in skeletal muscle. AMPK is activated under conditions of increased AMP/ATP ratio during muscle contraction and promotes metabolic adaptations including enhanced fatty acid oxidation, increased GLUT4-mediated glucose uptake and stimulation of mitochondrial biogenesis. These processes contribute to the metabolic adaptation of muscle to the energetic demands imposed by neural activity [243,244,245,246].

The organisation of motor units reflects this functional integration. Low-threshold motor units, characterised by tonic and sustained discharge patterns, predominantly innervate oxidative type I and type IIa muscle fibres, which exhibit high mitochondrial density and greater insulin sensitivity. In contrast, high-threshold motor units recruited during high-intensity activities predominantly innervate fast-contracting glycolytic fibres such as type IIx/IIb fibres [239,242]. Since skeletal muscle accounts for approximately 70–80% of insulin-stimulated glucose uptake, the organisation of the motor unit directly influences the efficiency of substrate utilisation and the maintenance of systemic glycaemic homeostasis [25,247].

### 6.3. Implications for Type 2 Diabetes and Metabolic Syndrome

Alterations in the motor neuromuscular axis have increasingly been recognised as a relevant component in the pathophysiology of chronic metabolic disorders, particularly type 2 diabetes mellitus and metabolic syndrome. These conditions are characterised by insulin resistance, low-grade metabolic inflammation and mitochondrial dysfunction, factors that directly affect both the structure and function of skeletal muscle and its motor units [241].

Individuals with type 2 diabetes frequently present structural alterations in skeletal muscle, including reduced muscle strength, decreased lean mass and changes in muscle fibre composition, with a relative increase in glycolytic fibres and reduction in oxidative fibres [7,243,248]. These alterations are accompanied by reduced mitochondrial density, impaired oxidative phosphorylation efficiency and decreased GLUT4-mediated glucose transport, processes that compromise efficient substrate utilisation and contribute to the development of insulin resistance [25,249,250].

In addition to intrinsic alterations in muscle tissue, evidence suggests that the structural integrity of the motor unit may also be compromised under metabolically dysfunctional conditions. Chronic hyperglycaemia, oxidative stress and systemic inflammation associated with diabetes may induce neuromuscular junction remodelling, reduced efficiency of synaptic transmission and progressive loss of motor units [215,251]. These alterations impair coordination of muscle recruitment and may contribute to the development of muscle weakness and to the establishment of a phenotype known as diabetic sarcopenia, characterised by progressive loss of muscle mass and function in individuals with diabetes [252].

From a metabolic perspective, deterioration of neuromuscular function may establish a pathological cycle in which reduced muscle activity and oxidative capacity decrease peripheral glucose uptake, thereby aggravating systemic insulin resistance and promoting loss of metabolic flexibility [230,253].

### 6.4. Integrative Conceptual Model

Based on the evidence discussed in this review, a conceptual model can be proposed in which the motor neuromuscular axis acts as a structural mediator between early metabolic exposures and the regulation of metabolic flexibility throughout life. In this model, alterations in the metabolic environment during critical developmental periods, such as maternal hyperglycaemia, nutrient excess or metabolic inflammation, may influence not only classical metabolic tissues but also the maturation of the neuromuscular system [41].

As discussed in previous sections, developmental processes involved in the establishment of the motor neuromuscular axis occur during critical windows of plasticity and may be influenced by metabolic and hormonal signals present in the foetal environment [36,67]. Alterations in these processes may affect the metabolic phenotype of skeletal muscle, particularly through changes in mitochondrial function, oxidative capacity and muscle fibre composition.

Considering that skeletal muscle represents the primary site of insulin-stimulated peripheral glucose uptake, changes in the functional organisation of the neuromuscular axis may compromise efficient energy substrate utilisation and reduce metabolic flexibility, a phenomenon frequently observed in conditions such as diabetes and metabolic syndrome [25,56]. Thus, the proposed model suggests that disturbances in the early metabolic environment may programme long-term alterations in the organisation of the neuromuscular system, influencing the metabolic capacity of skeletal muscle and contributing to susceptibility to metabolic diseases throughout life.

## 7. Knowledge Gaps and Experimental Directions

Despite significant advances in understanding the developmental origins of metabolic diseases, important gaps remain regarding the mechanisms linking early metabolic disturbances to the development and function of the neuromuscular system. The Developmental Origins of Health and Disease (DOHaD) paradigm has demonstrated that intrauterine exposures and events occurring during the first years of life can permanently programme multiple physiological systems. However, most investigations have focused on organs classically associated with energy homeostasis, such as the liver, pancreas and adipose tissue, while the role of the neuromuscular system in this process remains relatively underexplored. In this context, integrative approaches linking early metabolic exposures to structural and functional alterations of the motor neuromuscular axis throughout life represent a promising field of investigation.

### 7.1. Need for Longitudinal Studies Integrating Neurodevelopment and Metabolism

A large proportion of the available evidence derives from cross-sectional studies or isolated experimental models, which limits the understanding of causal relationships between early metabolic exposures and motor alterations across the lifespan. The DOHaD model proposes that events occurring during critical periods of development may permanently programme physiological systems, including energy metabolism and neuromuscular function. However, most investigations have concentrated on metabolic, cardiovascular or cognitive outcomes, with relatively few studies simultaneously integrating metabolic indicators and markers of motor neurodevelopment from the foetal period to adulthood [3,6,11,44].

Longitudinal studies are particularly important for identifying critical windows of vulnerability and plasticity, correlating metabolic parameters with neuromuscular maturation and distinguishing transient effects from persistent alterations. Prospective designs allow temporal relationships between exposure and outcome to be established, enable the identification of individual developmental trajectories and provide insight into how early metabolic disturbances influence functional development across the life course. Evidence from large birth cohorts indicates that adverse metabolic exposures during gestation and early childhood may produce long-lasting effects on metabolism, body composition and neuroendocrine function. However, few studies have simultaneously followed both metabolic and neuromuscular development over extended periods, highlighting an important gap in the current literature [11,254,255].

### 7.2. Epigenetic Evaluation of Motor Neurons

Another relatively unexplored area concerns the epigenetics of motor neurons. Experimental evidence from metabolic and neurobiological studies indicates that adverse metabolic states, including obesity, insulin resistance and hyperglycaemia, may induce epigenetic modifications such as DNA methylation, post-translational histone modifications and regulation mediated by microRNAs. These mechanisms modulate gene expression without altering the nucleotide sequence of DNA and may influence processes fundamental to neuronal function, including cellular differentiation, electrical excitability, axonal maintenance and neuromuscular communication [256,257,258,259].

Although these mechanisms have been extensively investigated in classical metabolic tissues such as liver, adipose tissue and skeletal muscle, studies specifically addressing motor neurons or the neuromuscular junction remain limited. Given the dependence of motor neurons on sustained electrical activity, high energy availability and trophic support, it is plausible that epigenetic alterations induced by adverse metabolic environments also influence neuromuscular function across the lifespan. However, this hypothesis is currently supported predominantly by evidence derived from neurodegenerative and neuromuscular disease models rather than studies specifically investigating early metabolic programming [256,257,258].

The epigenetic investigation of motor neurons presents important methodological challenges. Access to human neural tissue is limited by ethical and technical constraints, while the high cellular heterogeneity of the nervous system complicates the identification of alterations specific to neuronal subtypes. In this context, emerging technologies such as induced pluripotent stem cells (iPSCs), neuromuscular organoids and single-cell analysis techniques have emerged as promising tools for investigating molecular processes with greater resolution and physiological relevance [260,261].

### 7.3. Structural and Functional Analysis of the Motor Unit in Metabolic Programming Models

Despite advances in understanding the metabolic programming of tissues traditionally associated with energy homeostasis, the role of the neuromuscular system in developmental metabolic programming remains poorly understood. Most research within the DOHaD field focuses on organs such as the pancreas, liver and adipose tissue, whereas studies addressing the neural control of muscle function receive comparatively less attention.

However, skeletal muscle metabolism is largely regulated by the motor unit, which represents the smallest functional unit of the neuromuscular system. Each motor unit consists of a spinal motor neuron, its peripheral axon, the neuromuscular junctions and the group of muscle fibres it innervates. The physiological properties of these units directly influence muscle contractile capacity, fatigue resistance and the metabolic profile of muscle fibres [262].

During development, motor unit organisation emerges through highly regulated processes that include neuronal specification, axonal growth, formation of neuromuscular junctions and activity-dependent synaptic refinement. Initially, multiple axons may innervate the same muscle fibre, but a process of competitive synaptic elimination subsequently occurs until a single functional connection is established. This synaptic refinement takes place during critical developmental windows and is highly sensitive to molecular signals and patterns of neural activity [110,131].

Although substantial evidence demonstrates that the maternal metabolic environment influences overall neural development through inflammatory, hormonal and epigenetic mechanisms, whether these developmental perturbations directly affect the structural or functional organisation of motor units remains largely unknown. Most studies are limited to evaluating global muscular parameters, such as muscle mass or gene expression, without examining potential alterations in neuromuscular junction architecture, motor neuron identity or motor unit distribution. Investigations integrating morphological, electrophysiological and metabolic analyses may provide a more comprehensive understanding of how the neuromuscular axis contributes to metabolic programming.

### 7.4. Potential Future Experimental Strategies

Understanding the role of the neuromuscular axis in metabolic programming requires the integration of experimental approaches from developmental neurobiology, muscle physiology and metabolic biology. Recent advances in molecular and neurophysiological technologies have enabled the investigation of neural circuits and cellular trajectories with unprecedented resolution.

One promising strategy involves the use of single-cell transcriptomics to characterise populations of spinal motor neurons during development. This approach allows the identification of distinct neuronal subtypes and reconstruction of differentiation trajectories based on gene expression profiles. In parallel, animal models of metabolic programming, including maternal high-fat diets, nutritional restriction and experimental models of gestational diabetes, remain important tools for investigating causal mechanisms associated with the metabolic development of offspring [5,6,52,175].

In addition to structural approaches, functional analyses will be essential for understanding the impact of metabolic programming on the neuromuscular axis. Techniques such as in vivo electrophysiology, motor unit decomposition using high-density electromyography and optogenetic manipulation of motor neurons can provide direct measurements of neural activity controlling skeletal muscle. When combined with systemic metabolic assessments, including insulin sensitivity, muscle oxidative capacity and analyses of metabolic flexibility, these approaches will allow more direct testing of the hypothesis that early metabolic disturbances may remodel the structural and functional organisation of the neuromuscular system across the lifespan.

### 7.5. Sexual Dimorphism in Neuromuscular Metabolic Programming

Another important knowledge gap concerns the potential influence of biological sex on neuromuscular metabolic programming. A substantial body of evidence within the DOHaD framework demonstrates that males and females frequently exhibit distinct responses to adverse early-life environments, including differences in susceptibility to obesity, insulin resistance, type 2 diabetes and cardiovascular disease. These sex-specific outcomes are thought to arise from interactions among sex chromosomes, hormonal milieu and developmental timing, which collectively influence the programming of multiple physiological systems [263,264,265].

Sexual dimorphism is also evident within the neuromuscular system. Skeletal muscle mass, fibre type distribution, mitochondrial function and insulin sensitivity differ between males and females, while sex hormones contribute to the regulation of neuromuscular junction stability, motor neuron physiology and muscle plasticity. These observations raise the possibility that developmental programming of the motor neuromuscular axis may not occur uniformly between sexes and that similar metabolic exposures during critical developmental windows may produce distinct neuromuscular and metabolic consequences in male and female offspring [266,267].

Despite these observations, few studies have specifically examined whether maternal metabolic disturbances differentially affect the structural and functional development of the motor neuromuscular axis according to sex. Most experimental models either focus on a single sex or lack sufficient statistical power to evaluate sex-specific effects. Future investigations integrating neuromuscular, metabolic and developmental outcomes should therefore incorporate sex as a biological variable to determine whether sexually dimorphic mechanisms contribute to differential susceptibility to metabolic diseases across the lifespan [268,269,270].

### 7.6. Neuromuscular Plasticity and Lifelong Remodelling

Although developmental programming may establish long-term physiological trajectories, the motor neuromuscular axis remains highly plastic throughout life. Both neural and muscular components retain the capacity to adapt to environmental, metabolic and functional stimuli through processes involving synaptic remodelling, motor unit reorganisation and alterations in muscle phenotype. This plasticity provides the capacity for continuous adjustments in neuromuscular performance and metabolic function in response to changing physiological demands [127,251,270,271].

Skeletal muscle mass is dynamically regulated by the balance between protein synthesis and degradation. Hypertrophy results from anabolic processes that promote myofibrillar accretion and increased contractile capacity, whereas atrophy is associated with enhanced proteolysis, reduced muscle fibre size and impaired metabolic performance. These adaptations are influenced by neural activity, hormonal status, nutritional availability and systemic metabolic conditions. Because skeletal muscle represents the primary site of insulin-stimulated glucose disposal, alterations in muscle mass and quality have direct implications for whole-body metabolic homeostasis [238,272,273,274].

The existence of lifelong neuromuscular plasticity raises an important question within the DOHaD framework: to what extent can adverse developmental programming be attenuated, compensated or reversed by later-life interventions? Physical exercise is of particular interest in this context, as both aerobic and resistance training promote neuromuscular adaptations including motor unit remodelling, neuromuscular junction plasticity, mitochondrial biogenesis and improvements in muscle metabolic function. These observations suggest that the motor neuromuscular axis retains substantial capacity for functional reorganisation beyond developmental periods. However, it remains unclear whether exercise and other interventions can fully overcome neuromuscular alterations established during critical developmental windows or merely attenuate their long-term consequences. Future longitudinal and experimental studies should therefore investigate the extent to which programmed neuromuscular phenotypes remain modifiable throughout life and whether such interventions can alter susceptibility to metabolic diseases [275,276,277,278].

## 8. Translational Implications and Future Perspectives

### 8.1. Maternal Metabolic Control and Early Interventions

Maternal metabolic control during pregnancy represents a central factor in preventing alterations in foetal development. Metabolic conditions such as hyperglycaemia, dyslipidaemia, systemic inflammation and insulin resistance may modify the intrauterine environment and directly influence the development of multiple physiological systems, including the neuromuscular system [3,279]. Prolonged exposure to elevated levels of glucose, circulating lipids and inflammatory mediators may interfere with critical developmental processes such as motor neuron differentiation, myogenesis and neuromuscular junction formation [166,280].

Conditions such as gestational diabetes are associated with oxidative stress, metabolic inflammation and alterations in insulin signalling, factors that may contribute to an adverse intrauterine environment and influence the metabolic development of the offspring [182]. Chronic inflammation may in turn induce persistent immune responses resulting in continuous tissue damage and cellular remodelling, processes that may affect both muscle development and neural function [281,282].

In this context, adequate monitoring of maternal metabolic conditions becomes essential. Women with gestational diabetes mellitus present increased risk of gestational hypertension, foetal macrosomia and preterm birth. Strategies including glycaemic control, balanced nutrition, regular physical activity and specialised medical follow-up contribute to reducing foetal exposure to adverse metabolic alterations [283,284,285].

Beyond the gestational period, early interventions during the neonatal period and childhood may also exert long-lasting effects on metabolic health. Breastfeeding, for instance, is associated with improved metabolic regulation, reduced risk of obesity and increased protection against metabolic disorders throughout life. In parallel, adequate nutrition and appropriate motor stimulation during childhood contribute to the healthy development of the neuromuscular system and to the efficient regulation of energy metabolism [102,286,287,288,289].

### 8.2. Exercise During Pregnancy

Physical exercise during pregnancy has been increasingly recognised as an important non-pharmacological strategy for promoting maternal and foetal health. When performed safely and at moderate intensity, physical activity can improve maternal cardiovascular function, contribute to appropriate gestational weight gain and reduce systemic inflammatory processes [290,291,292].

From the foetal perspective, maternal exercise may increase placental blood flow and improve oxygen delivery to developing tissues, factors that support the growth and maturation of physiological systems such as the nervous system and skeletal muscle [293,294]. Moreover, regular physical activity during pregnancy is associated with a reduced risk of gestational diabetes and hypertensive disorders of pregnancy, conditions that can significantly alter the intrauterine metabolic environment [282,290,292].

These observations suggest that maternal physical activity may act as a positive modulator of metabolic programming in the offspring. Although the underlying mechanisms are not yet fully understood, experimental studies indicate that exercise-induced metabolic adaptations may influence placental function, nutrient availability and hormonal signalling during foetal development [6,52].

### 8.3. Pharmacological and Non-Pharmacological Treatments

When behavioural interventions are insufficient to maintain adequate metabolic control during pregnancy, pharmacological treatments may become necessary. In cases of gestational or pre-existing diabetes, insulin therapy represents one of the main therapeutic strategies, allowing effective glycaemic control without crossing the placenta in significant amounts. Pharmacological control of maternal glycaemia reduces foetal exposure to chronic hyperglycaemia and contributes to preventing metabolic alterations associated with the development of metabolic diseases later in life [295,296,297].

Nevertheless, non-pharmacological interventions continue to play a central role in the prevention of gestational metabolic disorders. Strategies such as balanced nutrition, nutritional counselling and regular physical activity adapted to pregnancy remain fundamental pillars for maintaining maternal and foetal metabolic health [282,289].

Maternal supplementation with n-3 polyunsaturated fatty acids, particularly those derived from fish oil, has also been investigated as a nutritional strategy capable of modulating the intrauterine metabolic environment. Compounds such as eicosapentaenoic acid (EPA) and docosahexaenoic acid (DHA) possess anti-inflammatory properties and may contribute to improving insulin sensitivity and regulating maternal lipid metabolism. In addition, DHA constitutes an important structural component of neuronal cell membranes during development, participating in processes related to neurogenesis, synapse formation and membrane functional organisation [14,298,299,300].

Experimental evidence suggests that maternal n-3 supplementation may attenuate systemic inflammatory processes, improve metabolic profiles in offspring and favour adaptations related to skeletal muscle oxidative capacity. However, the effects of such supplementation on metabolic programming and neuromuscular development remain incompletely established in humans, and further longitudinal studies and controlled clinical trials are required to clarify the role of these interventions during critical developmental periods [300].

### 8.4. Critical Windows for Metabolic Risk Prevention

The concept of critical windows refers to sensitive periods of biological development during which environmental, nutritional or hormonal exposures may influence future metabolic trajectories. The DOHaD paradigm emphasises that events occurring from the prenatal period to early childhood may permanently modulate physiological systems involved in energy metabolism [296,301,302].

Epidemiological studies demonstrate that inadequate nutrition during pregnancy or childhood is associated with increased risk of obesity, impaired glucose tolerance and hypertension in adulthood. Historical evidence derived from populations exposed to famine during gestation indicates that the timing of nutritional exposure may influence different metabolic risk profiles throughout life [303,304,305,306].

These observations suggest that nutritional and behavioural interventions during specific developmental windows may modify or even attenuate adverse effects of metabolic programming. Studies in animal models demonstrate that interventions applied during these periods of developmental plasticity may influence adipose tissue function, insulin signalling and energy metabolism, reducing characteristics associated with metabolic syndrome in adulthood [298,300,301,306].

### 8.5. Methodological and Regulatory Challenges

Investigating critical developmental windows and their relationship with metabolic risk presents important methodological challenges. A large proportion of available evidence derives from epidemiological and observational studies, which are essential for identifying associations between early exposures and metabolic outcomes but present limitations related to difficulties in establishing causality and the presence of confounding factors [307,308,309].

Longitudinal studies that follow individuals from pregnancy to adulthood are particularly complex, requiring large population cohorts and extended follow-up periods. Moreover, the heterogeneity of environmental exposures, including dietary patterns, socioeconomic conditions and psychosocial factors, complicates comparisons across studies and the precise identification of the most relevant determinants of metabolic programming [12,309].

Experimental animal models remain important tools for investigating biological mechanisms of metabolic programming, including epigenetic alterations, oxidative stress and hormonal dysregulation associated with metabolic syndrome [309]. However, extrapolating these findings to humans presents significant limitations, as differences in developmental timing and metabolic physiology across species may influence interpretation of results.

Another important challenge concerns the identification of reliable biomarkers capable of detecting metabolic programming at early stages. Although studies have identified epigenetic, metabolic and inflammatory alterations associated with early exposures, there is still no consensus regarding which markers present the greatest sensitivity and specificity for predicting future metabolic risk [56,309].

Overcoming these challenges will require integration of epidemiological, experimental and clinical studies, as well as multidisciplinary collaboration among fields such as endocrinology, neuroscience, nutrition and molecular biology. Progress in this area will also depend on the development of new technologies capable of monitoring molecular and metabolic processes across the life course, contributing to the translation of knowledge about metabolic programming into effective strategies for the prevention of chronic diseases.

## 9. Search Method Strategy

This manuscript was developed as a narrative review integrating evidence from the fields of developmental biology, neuromuscular physiology, metabolism and the Developmental Origins of Health and Disease (DOHaD) framework. The relevant literature was identified through searches of PubMed, Web of Science and Google Scholar using combinations of terms related to developmental programming, motor neurons, motor units, neuromuscular junctions, skeletal muscle metabolism, diabetes and metabolic syndrome. Articles were selected according to their relevance to the proposed conceptual framework and the objectives of the review.

## 10. Final Considerations

Over recent decades, the Developmental Origins of Health and Disease paradigm has substantially expanded our understanding of how early environmental exposures can influence health trajectories throughout life. Most of the accumulated evidence has focused on tissues classically associated with metabolic homeostasis, such as the liver, pancreas and adipose tissue, which play central roles in the regulation of energy metabolism. However, systemic metabolism results from dynamic interactions among multiple physiological systems, and approaches that consider this integration are essential for fully understanding the mechanisms linking early exposures to the risk of metabolic diseases.

In this review, we discussed evidence suggesting that the development of the neuromuscular system may represent a relevant structural component of metabolic programming. Evidence discussed throughout this review indicates that processes such as motor neuron differentiation, neuromuscular junction formation and the metabolic specialisation of skeletal muscle fibres may represent important targets of developmental programming during critical developmental windows. Because these processes are influenced by metabolic, hormonal and inflammatory signals present in the intrauterine and neonatal environments, alterations in the early-life environment may affect the structural and functional organisation of the motor neuromuscular axis. Considering the central role of skeletal muscle in glucose uptake and metabolic flexibility, such alterations may contribute to long-term changes in metabolic capacity and influence susceptibility to metabolic disorders across the lifespan.

The integration of motor neurodevelopment and metabolic programming therefore represents an emerging field of investigation with the potential to expand the conceptual framework of DOHaD. Future studies integrating structural and functional analyses of motor units, metabolic characterisation of skeletal muscle and molecular approaches to developmental biology may help clarify the mechanisms linking early metabolic exposures to the regulation of energy homeostasis. Progress in this field may not only deepen our understanding of the pathophysiology of metabolic diseases but also open new perspectives for preventive and translational strategies aimed at promoting health throughout the life course.

## Data Availability

No new data were created or analyzed in this study. Data sharing is not applicable to this article.

## Abbreviations

The following abbreviations are used in this manuscript:

ACh	Acetylcholine
AChR	Acetylcholine Receptor
AGEs	Advanced Glycation End-Products
AMPK	AMP-Activated Protein Kinase
ATP	Adenosine Triphosphate
CaMK	Calcium/Calmodulin-Dependent Protein Kinase
CD36	Cluster of Differentiation 36
CNS	Central Nervous System
CPT1A	Carnitine Palmitoyltransferase 1A
DHA	Docosahexaenoic Acid
DOHaD	Developmental Origins of Health and Disease
Dok7	Downstream of Tyrosine Kinase 7
EDL	Extensor Digitorum Longus
EPA	Eicosapentaenoic Acid
FGF	Fibroblast Growth Factor
FF	Fast Fatigable
FR	Fast Fatigue-Resistant
GABA	Gamma-Aminobutyric Acid
GDNF	Glial Cell Line-Derived Neurotrophic Factor
GLUT4	Glucose Transporter Type 4
HFD	High-Fat Diet
IGF-1	Insulin-Like Growth Factor 1
IL-6	Interleukin-6
iPSC	Induced Pluripotent Stem Cell
LMC	Lateral Motor Column
Lrp4	Low-Density Lipoprotein Receptor-Related Protein 4
MAPK	Mitogen-Activated Protein Kinase
MMC	Medial Motor Column
mTOR	Mechanistic Target of Rapamycin
MuSK	Muscle-Specific Kinase
NCDs	Non-Communicable Diseases
NFAT	Nuclear Factor of Activated T Cells
NMJ	Neuromuscular Junction
NT-3	Neurotrophin-3
PKA	Protein Kinase A
PKC	Protein Kinase C
PGC-1α	Peroxisome Proliferator-Activated Receptor Gamma Coactivator 1 Alpha
PI3K	Phosphoinositide 3-Kinase
PPARα	Peroxisome Proliferator-Activated Receptor Alpha
RAGE	Receptor for Advanced Glycation End-Products
RET	Rearranged during Transfection
ROS	Reactive Oxygen Species
SHH	Sonic Hedgehog
SOL	Soleus
T2DM	Type 2 Diabetes Mellitus
TCA	Tricarboxylic Acid Cycle
TLR4	Toll-Like Receptor 4
TNF-α	Tumour Necrosis Factor Alpha
Trk	Tropomyosin Receptor Kinase
UCP3	Uncoupling Protein 3
VEGF	Vascular Endothelial Growth Factor
Wnt	Wingless-Related Integration Site Signalling Pathway
